# The Integrase: An Overview of a Key Player Enzyme in the Antiviral Scenario

**DOI:** 10.3390/ijms241512187

**Published:** 2023-07-29

**Authors:** Gioele Renzi, Fabrizio Carta, Claudiu T. Supuran

**Affiliations:** Neuroscienze, Psicologia, Area del Farmaco e Salute del Bambino (NEUROFARBA) Department, Sezione di Scienze Farmaceutiche e Nutraceutiche, University of Florence, Via Ugo Schiff 6, Sesto Fiorentino, 50019 Florence, Italy; gioele.renzi@unifi.it (G.R.); claudiu.supuran@unifi.it (C.T.S.)

**Keywords:** integrase (IN) enzyme, allosteric IN inhibitors (ALLINIs), multimeric INIs (MINIs), IN-strand transfer inhibitors (INSTIs), strand transfer inhibitors, dual inhibitors, 3′-processing

## Abstract

Integration of a desossiribonucleic acid (DNA) copy of the viral ribonucleic acid (RNA) into host genomes is a fundamental step in the replication cycle of all retroviruses. The highly conserved virus-encoded Integrase enzyme (IN; EC 2.7.7.49) catalyzes such a process by means of two consecutive reactions named 3′-processing (3-P) and strand transfer (ST). The Authors report and discuss the major discoveries and advances which mainly contributed to the development of Human Immunodeficiency Virus (HIV) -IN targeted inhibitors for therapeutic applications. All the knowledge accumulated over the years continues to serve as a valuable resource for the design and development of effective antiretroviral drugs.

## 1. Introduction

The International Committee on Taxonomy of Viruses (ICTV) classifies the ribonucleic acid (RNA) containing viruses into groups III-VI based on genome pairing and sign [1,2,3]; additional taxonomic features are considered to discriminate each virus among the others [1,2,3]. A wide variety of RNA-based viruses, such as coronaviruses, influenzae, and HIVs, are well-known etiologic agents of recurrent infectious diseases in living organisms [3,4]. In addition to the structural differences among DNA- and RNA-containing viruses, their replication cycle makes them particularly interesting from the evolutionary viewpoint. For the purposes of this review, it is worth mentioning RNA viruses account for higher mutation rates, and that makes them very quick in adaptation to changing environments when compared to their DNA counterparts [5,6]. Such an aspect plays a crucial role when uncontrolled diffusion of viruses among human/animal/plant populations occurs, as it may easily turn into outbreaks or pandemics [6,7].

**Figure 1:**
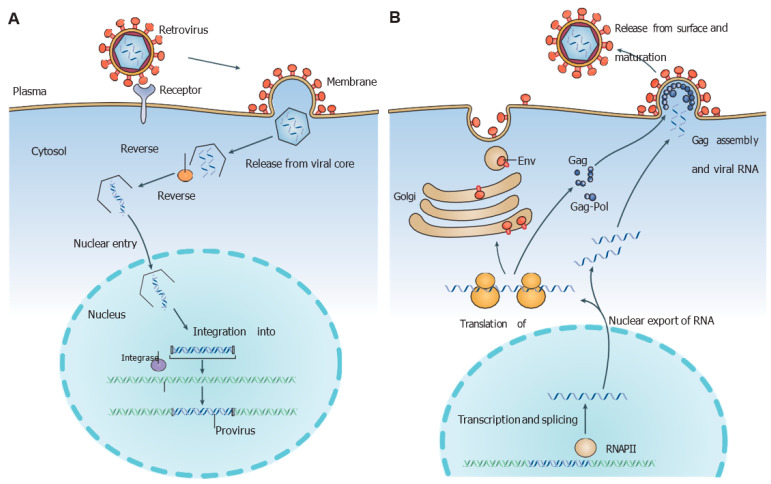
Replication cycle of retroviruses: (**A**) main steps for retrovirus entry within a eukaryotic cell and (**B**) for exit from it [5]. Figure was adapted with permission from Ref. [5].

All RNA viruses rely on their own “replicase complex” to replicate, which includes highly conserved enzymes associated with ancillary structural proteins. The RNA-dependent RNA polymerase (RdRp) is the most common enzyme packed within the replicase complex, although some viral strains may also contain RNA-helicases, NTPases as well as Integrases (IN) [4]. All such enzymes do catalyze key stages of the viral replication cycle, and for the purposes of this review, we focus on the integration of the viral double-stranded DNA molecule, transcribed from its RNA counterpart, within the host cell genome [5]. Reverse transcription (i.e., RNA to DNA) is a necessary step included in the replicative process of pathologically relevant retroviruses such as the human immunodeficiency virus 1 (HIV-1), murine leukemia virus (MLV), and avian sarcoma leukosis virus (ASLV). Figure 1A,B reports the most important replicative steps for such viruses.

In this context, the viral Integrase (IN; EC 2.7.7.49) enzyme plays a fundamental role as it efficiently mediates the integration of the newly transcribed DNA genome into the infected host cell. In this review article, the authors report fundamental knowledge on the IN enzymes and, unless otherwise stated, specifically refer to the HIV-1 expressed isoform, which is the best characterized so far and thus generally considered as a reference among the scientific community. A selection of the main scientific contributions dealing with the IN enzyme catalytic mechanism, structural aspects, and its role in the management of viral RNA-promoted infection diseases will be discussed. The final section will be dedicated to the most innovative and promising patents covering the last 10 years.

## 2. Structure of the IN Enzyme

**Figure 2:**
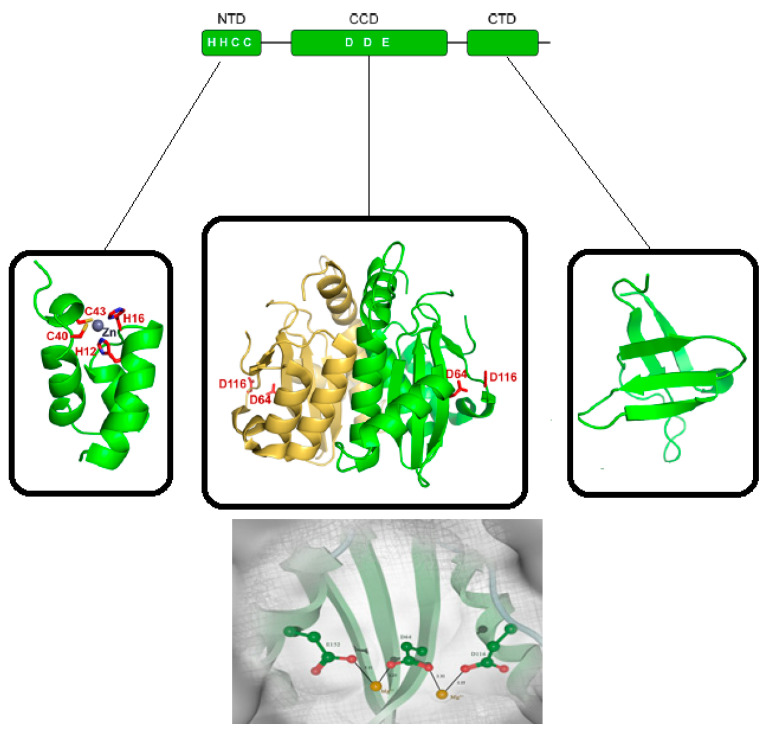
A schematic diagram of the three domains in HIV1-IN (**top**); structural representation of the NTD, CTD, and CCD (**middle**); HIV-1 IN catalytic core containing the DDE motif (**bottom**).

IN is a member of the tyrosine recombinase family, composed of the highly conserved structural domains referred to as the *N*-terminal domain (NTD), *C*-terminal domain (CTD), and catalytic core domain (CCD), which are schematically depicted in Figure 2. All three IN domains have been characterized in detail by NMR and X-ray crystallographic experiments [8,9,10,11,12,13,14].

A representative feature of the NTD-IN is the highly conserved HHCC motif useful for binding to the viral DNA and cleaving its strands. The domain is formed by three compact α-helices stabilized through coordination with a Zn (II) ion [13,14]. The length of NTDs is variable among retroviruses, such as in ε- and γ-retroviral spumaviral INs, which extends up to ~40 amino acid residues when compared to the HIV-1 expressed isoform [15].

**Figure 3:**
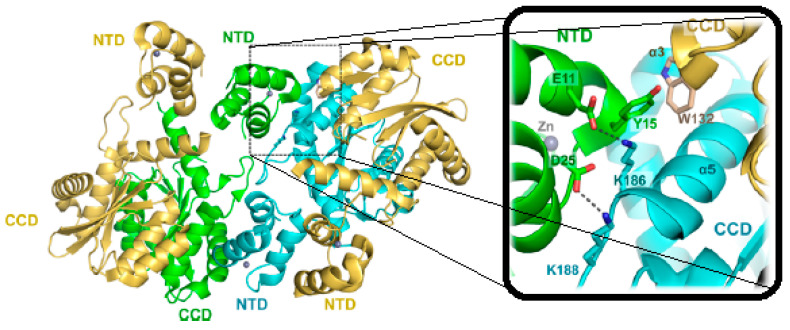
Tetrameric organization of HIV1-INs based on CCD dimerization and details of the key contacts at the CCD-NTD interface (PDB ID 1K6Y).

The CCD domain contains the IN active site with the highly conserved DDE motif, which is responsible for coordinating two Mg (II) ions [16] (Figure 2). Interestingly, X-ray crystal studies on the CCD-IN section revealed a protein folding typically contained in Nucleotidyl Transferases (NTs) [12] (Figure 3).

The large spatial separation between the enzyme active site, located at the CCD domain, from both the NTD and CTD sections is indicative of a high-level of multimeric organization for the entire IN enzyme [12]. X-Ray crystallographic experiments on the CCD-IN section partially confirmed such a hypothesis since most of the structures were solved as dimers [12]. The necessary multimeric functional organization of IN turns out to be particularly challenging to clarify. Although high aggregation states have been reported [17,18], the tetrameric assembly of the HIV-1 IN has received the strongest experimental support [19,20]. Studies on variegated retroviral genera predicted specific functional multimeric aggregations for their INs [21,22,23,24]. Overall, it is sufficient to summarize that any functional IN multimeric aggregation relies fundamentally on the dimerization of the CCD domains, which in turn are interconnected to each other at the NTD-CCD level, as shown in Figure 3.

Among the three IN domains, CTD is the least conserved. Nevertheless, important features such as β-folding of Src homology 3 (SH3) are highly conserved [8,9]. In addition, there is a closely related organization typically recovered within the Tudor family domains, which are known to be involved in chromatin binding processes [8,9].

During viral genome integration, both CTD and NTD are critically involved in docking and blocking the viral DNA (*v-*DNA*)*-substrate [25,26]. The high degree of flexibility of the linkers connecting the CDD to the outer domains (i.e., NTD and CTD) appears to vary widely in length among retroviruses and turns out to be particularly important for ensuring correct docking of the *v*-DNA [16,27].

Once host cells are infected, the pool of INs is mainly localized at the cytoplasm level as part of the viral pre-integration complex (PIC). PICs are rather complex units highly variable among viral strains, which are formed by the assembly of enzymatic and structural proteins. In acute viral infections, a single copy per cell usually is recovered, and that contributes to making PICs very difficult to identify and characterize [28,29,30,31,32,33,34,35,36,37].

The complete integration of the viral genome within the cellular host occurs through a finely regulated mechanism based on the activity of PICs once they have access to the cell nucleus. The IN enzyme inserts *v*-DNA into cell chromosomes, and the process is subsequently completed by the host cell’s DNA repair machinery [29,30,31,32,33,34,35,36,37]. The minimal substructure within the PIC that catalyzes viral genome integration in vitro is referred to as the Intasome (INT), which is highly specific for each retrovirus and its mutant strains [28,29,30]. To date, X-ray crystallography and cryogenic single-particle electron microscopy (cryo-EM) experiments made it possible to decipher the level of complexity of various INTs and turned very useful for the design of molecular scaffolds potentially useful for biomedical purposes [28].

## 3. Reactions Catalyzed by the IN Enzyme

**Figure 4:**
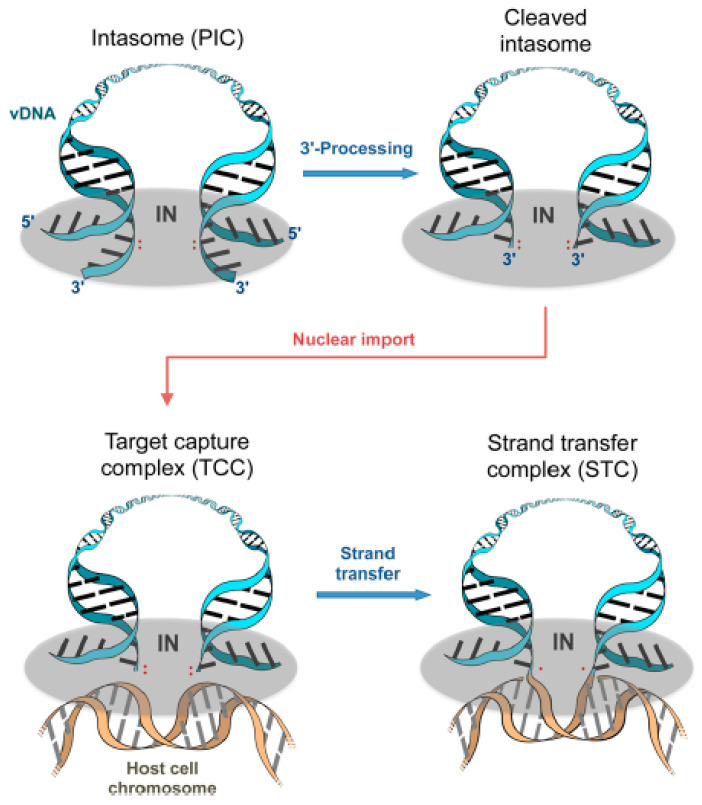
Integrase mechanisms.

Two main reactions are associated with the IN enzyme within the PICs, namely the 3′-OH processing (3-P) and the strand transfer (ST) [38,39,40,41], and are schematically reproduced in Figure 4.

The first stage of the mechanisms takes place at the cytoplasmic level, with the IN being part of the PIC. The enzyme first recognizes and binds the *v*-DNA at specific recognition sites and then cleaves the *v*-DNA strains creating a 3′-OH group that is available for nucleophilic attack. Next, the PIC translocates into the cell nucleus, and the IN binds to the host DNA at definite attachment sites and makes use of the newly generated 3′-OHs on the *v*-DNA to attack the host DNA strand at the phosphodiester bond between the 3′-OH and the 5′-phosphate. This results in an effective connection of the *v*-DNA into the host genome.

Since the 3′-processing step takes place at the cytoplasmic level, it is reasonable to expect that PICs will be surrounded by elevated local concentrations of linear *v*-DNAs. Such a situation may potentially represent a problem for retrovirus replication as self-integrating end-products could be generated. Such an effect is routinely observed in laboratory experiments, and it has been widely reported in in vivo cellular models of infection [42,43,44]. Interestingly, several retroviruses have developed very efficient strategies to avoid self-integration of their genomes, thus making their replication cycles more efficient. One example is the expression of the highly conserved cellular protein called the Barrier-to-Autointegration Factor (BAF). BAF is a small DNA-binding protein capable of bridging and condensing separate DNA molecules [45,46]. In vitro experiments on murine leukemia virus (MLV), PICs clearly showed the effects of BAF on self-integration events [47]. Although BAF showed an excellent ability to inhibit the self-integration genome in vitro, it is yet to be determined whether it acts effectively during MLV-promoted infections in in vivo models [47].

Alternative processes to prevent self-integration have been identified. It was reported that self-integration during HIV-1 infection is suppressed by the SET complex, an endoplasmic reticulum-associated complex containing three distinct DNase enzymes [44,48]. Overall, different retroviral species have evolved unique ways to protect themselves from suicidal genome integration events.

The occurrence of aberrant STs (i.e., half-site integration process) seems particularly relevant to functional PICs in vitro, whereas such events are scarcely reported when naturally occurring infections take place [49,50,51,52,53,54,55,56]. In addition to the 3-P and ST IN-associated activities, the reversal reaction of genome integration, referred to as “disintegration”, is known to be catalyzed by IN in vitro, but it has never been reported in vivo [57,58,59,60,61,62,63,64,65,66].

**Figure 5:**
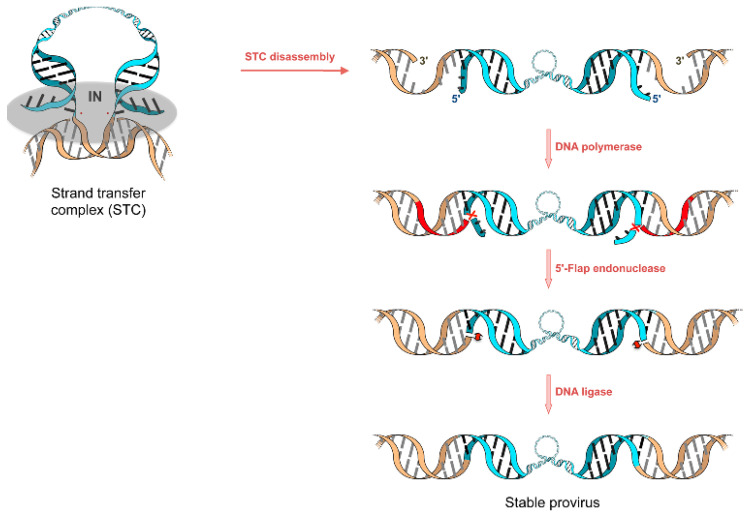
Before DNA repair enzymes are engaged, the dismantling of the highly thermodynamically stable strand transfer complex (STC) is necessary. This event is critical to ensure successful viral replication, although it has still been marginally considered being only one scientific study reported to date [67]. Specifically, the authors demonstrated that the proteasome-mediated degradation of HIV-1 IN involves the von Hippel–Lindau protein 1, resulting in exposure of DNA to the activities of a DNA polymerase, a 5′ lamella endonuclease, and an enzyme ligase (Figure 5). A putative role of IN in post-integration DNA repair has been hypothesized [57,68,69], but evidence for its efficacy in infection models is still lacking.

Once the ST process is complete, the new genome contains only two newly formed junctions which ensure the physical integrity of the *v*-DNA in the host. Therefore, a series of DNA repairing activities do take place with the final aim of fixing a pair of single-stranded gaps and two short 5′ overhangs *v*-DNA remaining sections (Figure 5).

## 4. IN-Strand Transfer Inhibitors (INSTIs)

To date, the combination antiretroviral therapy (cART) accounts for the association of four distinct classes of drugs: (i) nucleoside reverse transcriptase (RT) inhibitors (NRTIs) (ii) non-nucleoside RT inhibitors (NNRTIs); (iii) protease (PR) inhibitors (PIs), (iv) and the last introduced, the IN-strand transfer inhibitors (INSTIs).

A real breakthrough in cART therapy was achieved with the introduction of INSTIs into the clinic, as these drugs are the only ones capable of targeting a specific process that is exclusive and essential for retroviruses (i.e., viral genome integration) [70].

**Figure 6:**
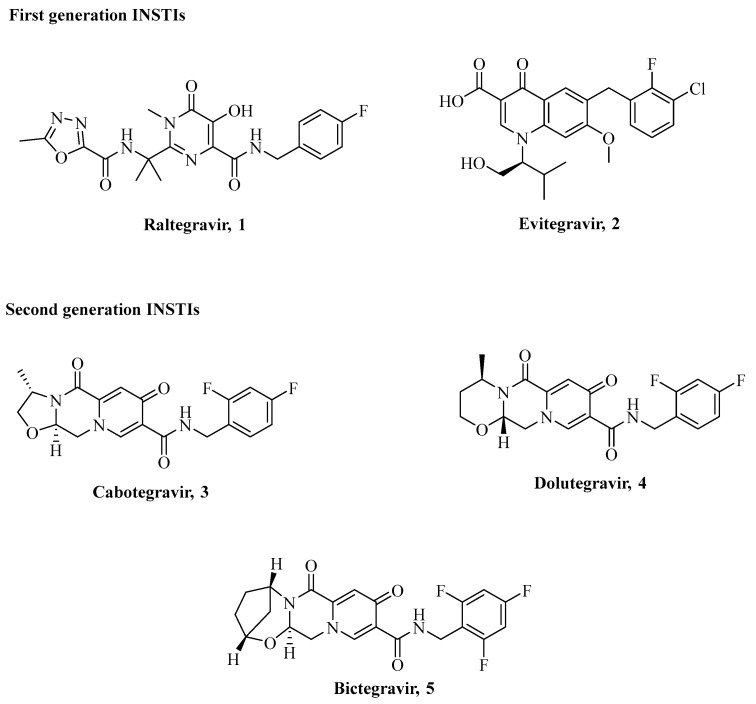
Chemical structures of clinically relevant INSTIs **Raltegravir 1**, **Elvitegravir 2**, **Cabotegravir 3**, **Dolutegravir 4**, and **Bictegravir 5**.

HIVs directed cART within the timeframe 1990s-early 2000s was represented exclusively by NNRTIs, NRTIs, and PIs. Multidrug and cross-drug resistances, mainly attributed to specific mutations on target proteins, led to a drastic reduction of cART clinical efficacy. The discovery of HIV-IN and the development of appropriate and reliable in vitro small molecule screening assays appeared when the loss of cART efficacy was seriously compromised, thus with a tangible risk of a pandemic at a global scale. The chemical structures of currently used INSTIs are depicted in Figure 6.

**Figure 7:**
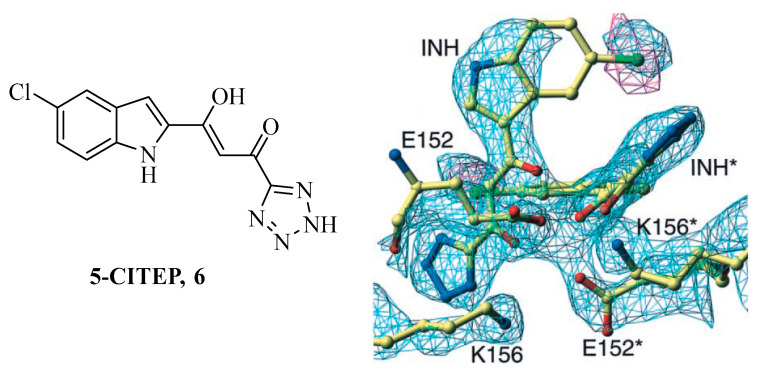
Chemical structure of the experimental INSTI **5-CITEP** (**6**) and electron density maps of the IN CCD active site region in complex with it [72].

Despite the classification in two generations, all clinically used INSTIs are structurally related to the experimental compounds 1-(5-chloroindol-3-yl)-3-hydroxy-3-(2*H*-tetrazol-5-yl)propenone **6** (**5-CITEP**) as do share the key diketoacid (DKA) moiety (Figure 7) [71,72,73].

Compound **6** is an α,γ-DKA with the carboxylic acid function replaced by the acidic bioisosteric tetrazole ring. The ligand showed IC_50_ experimental values of 0.65 μM for the ST and 35 μM for the 3-P, respectively [71]. It was the prototypic IN inhibitor successfully crystallized in adduct with the CCD domain [72]. Electron density maps accounted for its positioning among CCD residues D64, D116, and E152. Additional contacts of compound **6** with CCD residues responsible for docking the host DNA were also retrieved.

**Figure 8:**
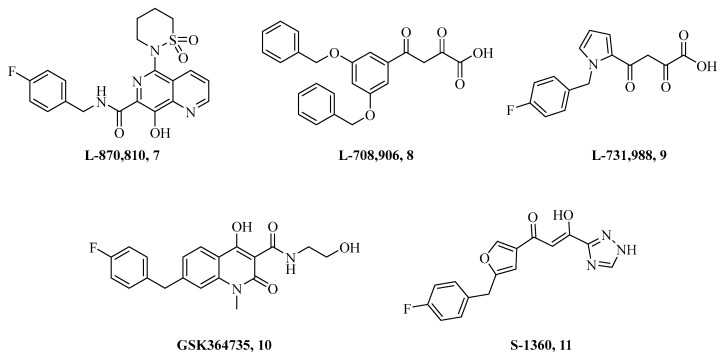
Chemical structures of DKA compounds **7**–**11**.

Parallel research in this field enabled Merck to discover **L-708,906** [74] and **L-731,988** [75] (i.e., compounds **8** and **9** in Figure 8), with the latter being particularly potent in inhibiting HIV1-IN in vitro with remarkable selectivity for the ST process over 3-P (IC_50_ of 80 nM and 6 μM, respectively) [74,75]. Remarkable results on infected cells have been obtained for the series of DKA compounds containing the 8-hydroxy-[1,6]-naphthyridine-7-carboxamide moiety, and among them, the derivative **L-870,810** (**7**) was found to be a potent inhibitor of IN at the ST level (IC50 of 8 nM) and was endowed with effective antiviral features on cell-based assays (i.e., EC_95_ =15 nM) [76]. Compound **7** was the first INSTI to show anti-HIV activity in experimental animal models and was, therefore, the subject of clinical trials, which, however, failed due to liver and kidney toxicity in dogs [77,78]. Better results were obtained with the DKA derivative (*Z*)-1-[5-[5-(4-fluorobenzyl)furan-2-yl]-3-hydroxy-3-(1H-1,2,4-triazol-3-yl)propenone **S-1360** (**11**), jointly developed by GlaxoSmithKline (London, UK) and Shionogi (Osaka, Japan). Compound **11** showed an IC_50_ in vitro value of 20 nM for the ST associated with highly effective suppression of HIV replication in in vivo infected models [79]. Preclinical assays on compound **11** were satisfactory, and in humans, it was demonstrated to be subjected to rapid metabolism and clearance through a non-CYP450-mediated pathway [80]. The joint venture between GlaxoSmithKline and Shionogi was also successful in developing the 4-hydroxy-2-oxo-1,2-dihydro-1,5-naphthyridine **GSK364735** [81] (i.e., compound **10** in Figure 8), which was revealed a remarkable INSTI and very effective in suppressing viral replication in cellular assays [81]. Unfortunately, long-term safety studies on monkeys reported severe hepatotoxicity for this compound that was discontinued from clinical development [82].

The intimate mechanisms of IN for both 3-P and ST catalyzed reactions were evaluated in detail through all compounds above-mentioned, which turned out to be of great value in the field of research, although they failed to enter the clinical armamentarium.

The compounds in Figure 7 and Figure 8 strongly contributed to elucidate that: (i) stable, functional PICs are most likely assembled using two Mg (II) ions during the *v*-DNA integration process; (ii) of the two metal ions, one appears to be coordinated by residues D64 and D116, while the second is involved in coordination by residues D116 and E152 [83,84]; (iii) DKA-containing molecules characterized by a γ-ketone, an enolizable α-ketone, and a carboxylic acid moiety result in effective inhibition of the IN enzyme. Acidic bioisosteric functional groups, such as tetrazole and triazole, or basic ones (i.e., pyridines) can be considered instead [83,84,85], thus suggesting that the potent antiviral ability of DKAs or their surrogates is well maintained as long as the chelating properties towards divalent metal ions are not disrupted [83,84,85]. Large series of mono-DKAs, dimeric DKAs, and triketoacids (TKAs) have been largely explored for their role in chelating divalent metal ions and proved to be potentially useful for IN inhibition by interfering with their supramolecular organization [86]. Overall, moderate IN inhibition potencies, much lower than those of DKAs, have been reported from both dimeric DKAs and TKAs [87].

## 5. First-Generation INSTIs

The first INSTI for the treatment of HIV-1 infections was approved by the US FDA in 2007 [88,89,90,91]. **Raltegravir** was the unexpected and relevant result of a wide R&D program intended to find HCV polymerase inhibitors based on small molecules containing the DKA moiety. **Raltegravir** proved to be potent, reversible, and selective INSTI with an IC_50_ value of 0.085 μM [92], highly effective in clinical trials when administered orally at a dosage of 400 mg twice/day with associated good tolerability and safety profiles and were devoid of any significant drug interactions [88,90,93,94,95]. Viral strains carrying mutations in the IN-CCD domain have appeared rapidly due to the wide use of **Raltegravir** since its approval. The most common mutations are E138A/K, Y143C/R, T66A, G140A, Q148/H/K/R, N155H, and Q95K [92], three signature resistant-associated mutations N155H ± E92Q, Q148H/K/R ± G140S/A and Y143C/R ± T97A accounted for a 10-fold reduced susceptibility to **Raltegravir** [90], while E138A, G140A and Q148K were identified as responsible for reduced drug susceptibility up to several hundred-folds [90].

FDA approval for the management of HIV-sustained infections was also obtained for **Elvitegravir**. This compound was initially developed by the Central Pharmaceutical Research Institute of Japan Tobacco, Inc. (Osaka, Japan) and later licensed to Gilead Sciences (Foster City CA, USA) for clinical development. **Elvitegravir** makes use of the 4-quinolone-3-carboxylic acid and is chemically derived from quinolone-type antibiotics. It showed very potent IN inhibitory activity for ST with an IC_50_ value of 7.2 nM, and even more interesting are the results obtained on acute HIV-1 infection assay, which showed **Elvitegravir** being active with an EC_50_ of 0.9 nM [96,97]. Unfortunately, cross-resistance events due to mutations towards **Raltegravir** and **Elvitegravir** were reported, thus reducing the chances of drug switching when cART therapeutic protocols are considered. United States and UE have licensed **Elvitegravir** as single table formulations named STRIBILD containing the pharmacokinetic enhancer cobicistat (COBI) able to inhibit the CYP3A4 enzyme, tenofovir disoproxil fumarate (TDF) and the NRTIs emtricitabine (FTC) [98]. Furthermore, a significant reduction in bone and renal side effects compared to STRIBILD was shown with the approval of the lower-dose single-tablet regimen Genvoya [99].

## 6. Second-Generation INSTIs

This section reports INSTIs compounds clinically licensed for therapeutic use, whereas experimental compounds currently undergoing clinical trials at different stages are not discussed as their chemical characteristics and preliminarily disclosed biomedical data largely overlap with known art. Second-generation INSTIs are characterized by chemical structures able to completely occupy the IN active-site regions.

Shionogi and Glaxo Smith Kline jointly discovered and developed **Dolutegravir** [100,101], which was marketed from the former as a 50 mg tablet under the brand name Tivicay R^®^. The genetic cross-resistance observed for the first-generation INSTIs Raltegravir and **Elvitegravir** found an effective replacement with the introduction of **Dolutegravir**. The optimization of a series of carbamoyl pyridone analogs which do retain a two-metal chelation ability within the IN catalytic active site, is directly linked to the chemical strategy underlying the effect of **Dolutegravir** [101]. Tricyclic carbamoyl pyridine is explained, through important structure-activity relationships (SARs), as an essential group that shares its oxygen-derived lone pairs to coordinate the two divalent metal ions within the IN active site [101]. This implies that the metal coordination of **Dolutegravir** doesn’t directly involve the carbonyl at the 5-positioned carboxamide moiety. As a result of such enhanced structural flexibility, this drug shows better embedment within the enzyme active site, and that improved its response to IN structural changes due to mutations typically triggered by exposure to first-generation INSTIs [102].

Shionogi-ViiV Healthcare and GSK developed **Cabotegravir** which is structurally related to **Dolutegravir** [103]. This drug was licensed by the US FDA in the fall of 2021 as a prophylaxis agent [104]. **Cabotegravir** showed an improved pharmacokinetic profile [105], a superior genetic barrier to resistance when compared to **Dolutegravir**, and, most importantly, a half-life of about 30 h [105,106]. Parenteral administration of **Cabotegravir** in nanosuspensions (i.e., Apretude^®^) allows single administration at low dosages once per month [107].

A genetic barrier to resistances superior to both first-generation INSTIs and **Dolutegravir** was reported by the potent INSTI **Bictegravir** (i.e., IC_50_ of 7.5 nM), which was approved in 2018 [108,109]. **Bictegravir** showed synergistic antiviral effects in vitro when combined with the N/NRTIs tenofovir alafenamide (TAF), emtricitabine (FTC), or PI Darunavir [109]. The single tablet Biktarvy ^®^ combining **Bictegravir** with FTC and TAF [110] was developed through the contribution of Gilead Sciences.

## 7. IN Inhibition: New Perspectives

### 7.1. Allosteric IN Inhibitors (ALLINIs)

Despite the great advantages brought to cART therapy from the introduction of INSTIs, clinical reports account for a multitude of drug resistance events. The interactions occurring between IN and cellular co-factors necessary for the PICs/INTs to be functionally active may be intentionally altered by the use of allosteric inhibitors (ALLINIs). All compounds classified as ALLINIs bind to IN regions distinct from the catalytic site and determine inhibition/disruption of the enzymatic activities [111,112,113].

ALLINIs do promote the formation of stable and high-order multimeric IN associations devoid of any catalytic properties. There remains scarce related knowledge regarding the mechanisms underlying ALLINIs’ mode of action, and detailed structural information for these aberrant IN multimerization complexes does appear very general. The important aspects to consider are the following: (i) for certain chemical classes of ALLINIs, any specific aberrant enzymatic multimerization is consistently observed; (ii) the functional dynamic flexibility between IN subunits is strongly affected by this unnatural, although thermodynamically very effective, structural organization [114].

The following are the most important ALLINIs.

### 7.2. Lens Epithelium-Derived Growth Factor/p75 (LEDGF/p75)

LEDGF/p75 is among the first discovered essential cellular components able to enhance the interaction of the host DNA with functionally active PICs. It binds through coordination with residues Asp366, Val 408, Ile365, and Phe406 to a specific site located at the IN-protein *C*-terminus, referred to as the integrase binding domain (IBD) [115,116,117].

**Figure 9:**
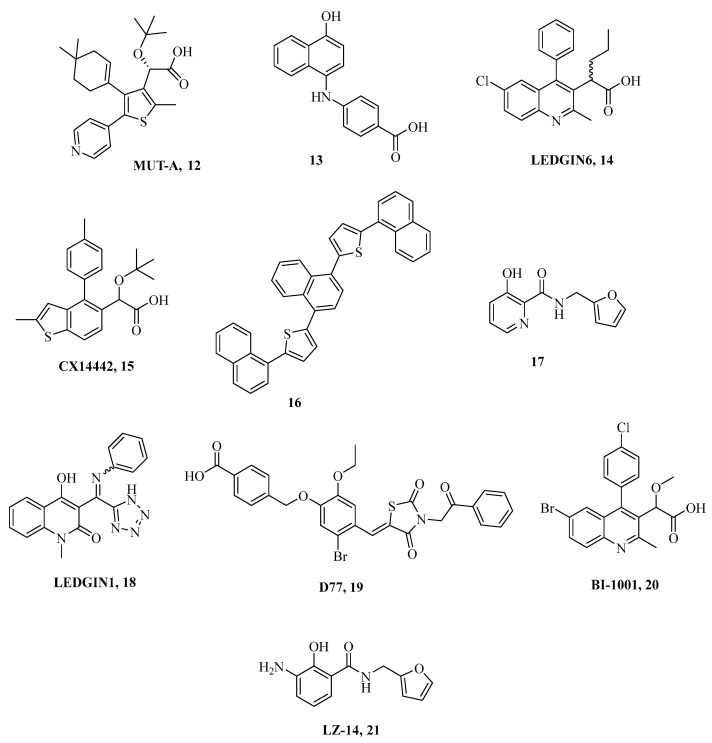
Chemical structures of the most representative LEDGINs.

Specifically, targeting the LEDGF/p75 interaction site affects the IN multimerization status, and thus, it allosterically affects its activity [117,118]. In addition, LEDGINs are also responsible for inducing altered catalytic activity of the enzyme with poor discrimination between ST and 3-P [119]. Since LEDGINs and INSTIs act through different mechanisms of action on ST catalytic activity, no cross-resistance events take place [120]. Notably, the road for cART therapy, including LEDGIN/INSTIs, has been opened by reporting that both act in an additive or synergistic way. The structures of the most promising LEDGIN compounds are shown in Figure 9.

### 7.3. Multimeric INIs (MINIs)

**Figure 10:**
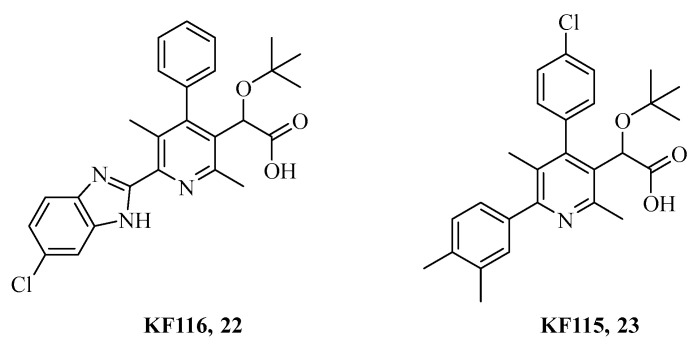
Chemical structures of the most representative MINIs.

Multimerization-selective INIs (MINIs) represent a distinct class of ALLINIs, as do result ineffective in the early steps of viral replication while do act only during the virion maturation steps by inducing aberrant IN multimerization. **KF115** and **KF116,** which were obtained by the introduction of the quinolone of **BI-1001** into the biaryl pyridine moiety instead, are the most advanced experimental compounds to date and era reported in Figure 10 [121,122].

## 8. Dual Acting Inhibitors

The development of dual-acting inhibitors, thus single molecules able to simultaneously modulate different and/or multiple targets with additive or synergistic therapeutic fashion, represent new advancements in the field of IN modulators for the management of retrovirus-promoted infections [123].

The application of these inhibitors in the clinic is expected to induce much more efficient therapeutic outcomes, possibly associated with increased patient compliance and fewer side effects. This strategy is adopted to target primarily key enzymes of the viral replication cycle. The most promising chemical classes are reported below.

### 8.1. IN-RT RNase H Inhibitors

The most immediate approach is represented by dual-acting inhibitors, which target both the IN and the RT-RNase-H enzymes since both share a common structural site composed of a central five-stranded mixed β-sheet next to α-helices [124,125,126]. Moreover, the potential action of chelating two magnesium metal ions in their active sites by the two enzymes is carried out through the presence of key acid amino acids (i.e., D443, E478, D498, and D549 for the RNase H domain and D64, D116, and E152 for HIV-1 IN) [126]. It is possible that DNA aptamers may possess inhibitory activity for IN, in analogy to DKAs, due to this close structural similarity [127].

### 8.2. INI-LEDGF/p75-IN Interaction Disruptors

**Figure 11:**
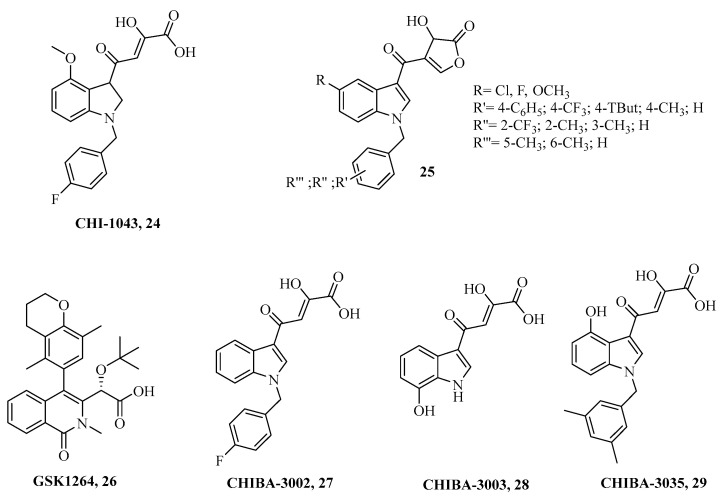
Chemical structures of IN-LEDGF/p75-IN disruptors.

Figure 11 reports experimental compounds possessing INI activity and disruption of the LEDGF/p75-IN interaction.

It was reported, by means of in silico studies, that the putative mechanisms of compound **24** bind the IN enzyme by coordination of the two metal cofactors, thus similarly to known INSTIs bearing the DKA moiety. Enzymatic experimental assays on compound **24**, its derivatives as well as their metal complexes proved inhibition of the IN-associated ST catalytic activity is in agreement with previous data with EC_50_ values spanning between nano- to micromolar range. The interaction between IN and LEDGF/p75 was disrupted by both the metal complexes and their free ligand counterparts when administered at low micromolar ranges. Overall, the Mg (II) complexes were far more attractive since data on infected cells showed quite interesting antiviral features [128]. Merge of the CHIBA series with CHIs scaffold afforded derivatives of the general structure **25** in Figure 11. Such an approach aims to create a molecular entity that combines the experimentally reported activity of compound **24** to affect either the IN ST process and the IN-LEDGF/p75 interaction [129] with CHIBAs compounds **27**, **28,** and **29** to inhibit the association of LEDGF/p75 to IN [130]. Dual action for the LEDGF/IN association as well as 3-P IN activity, also appears to be associated with compound **26**. Very interestingly, this compound prevents new virions from infecting host cells by acting on late stages of viral replication (i.e., post-integration) [131]. X-ray crystallization experiments assessed the binding mode of compound **26** within the IN and clearly showed it binds to the α1 and α3 helices of the first IN monomer subunit and to the α4 and α5 helices of the second monomer [131].

## 9. Integrase Inhibitor: Current Knowledge about Therapies

In this section, we report a selection of the most promising patents claiming IN inhibitors are able to reduce the progression of the viral infection and/or to reduce transmission of the virus.

**Figure 12:**
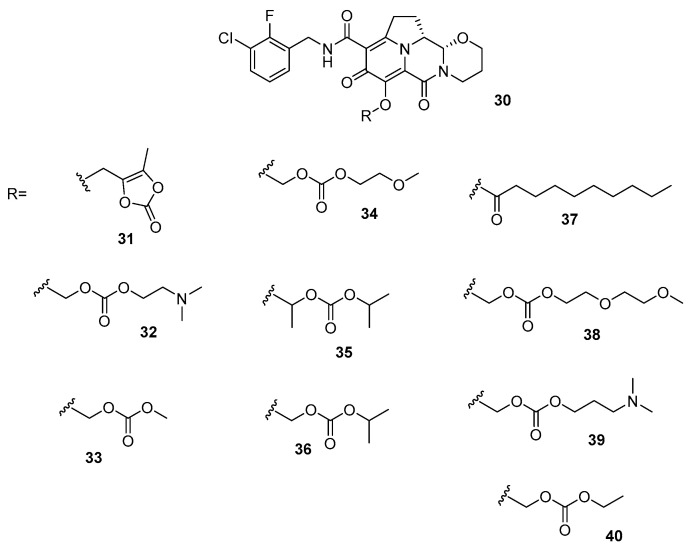
General structure of tetracyclic heterocyclic compounds **31**–**40** [132].

The first invention claims the use of tetracyclic heterocyclic compounds of the type reported in Figure 12 [132].

**Figure 13:**
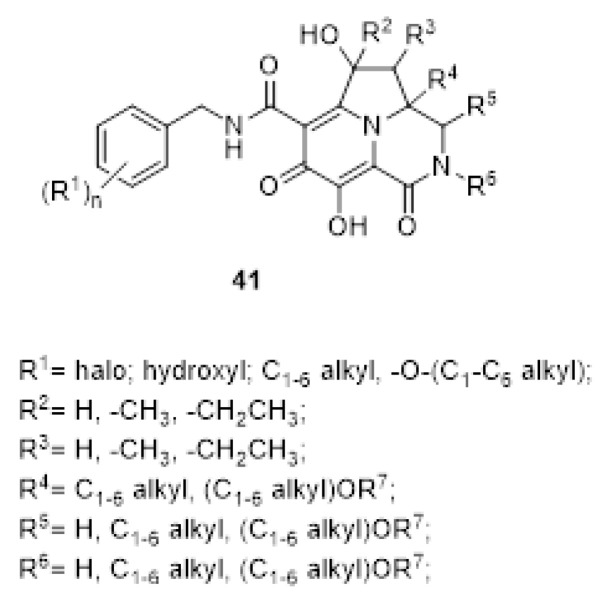
General structure of tricyclic heterocyclic compound [133].

Such an invention is followed by a related patent which claims tricyclic heterocyclic derivatives instead (Figure 13) [133].

Either the tetracyclic and the tricyclic derivatives in Figure 12 and Figure 13 are claimed as useful inhibitors of HIVs by targeting their IN enzymes [132,133] and thus useful for the management of HIV infections and/or reduction of the severity of symptoms of HIV infection in cell-based systems [132,133].

**Figure 14:**
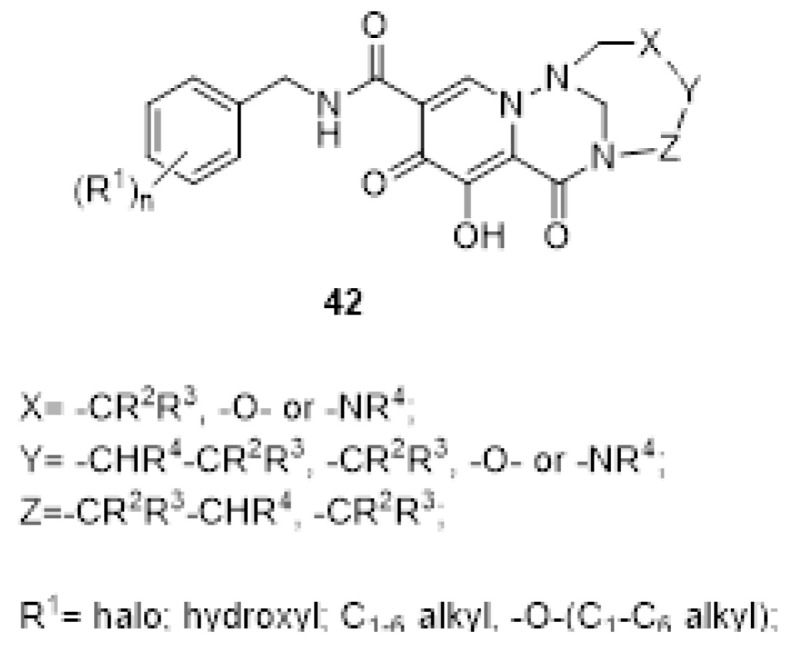
Tricyclic derivatives by modification of **41** [134].

The tricyclic compounds of general scaffold **41** can be modified, as reported in Figure 14, to afford the series **42**, which maintained the same therapeutics indications and efficacy against HIV-promoted viral infections [134].

**Figure 15:**
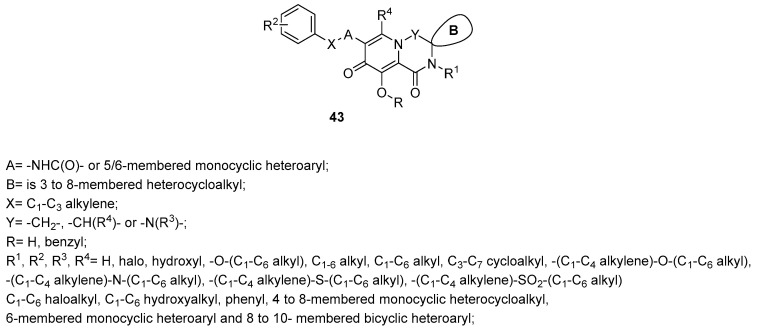
Spirocyclic pyridotriazines [135].

**Figure 16:**
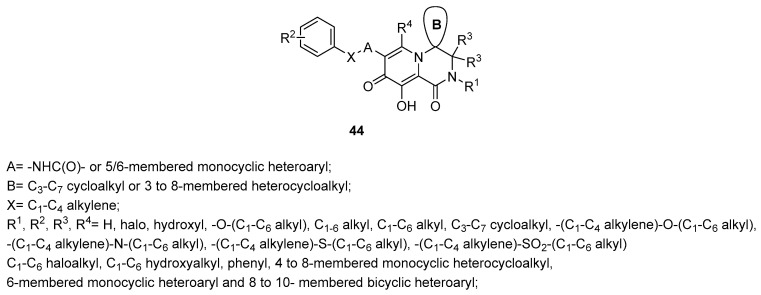
Spirocyclic heterocycles **44** [136].

The spirocyclic pyridotriazines of the general structure **43** in Figure 15 [135] or more, in general, the spirocyclic heterocycle derivatives of the type **44** in Figure 16 [136] were all claimed as inhibitors of the IN enzyme with interesting data in cellular model of the infection.

**Figure 17:**
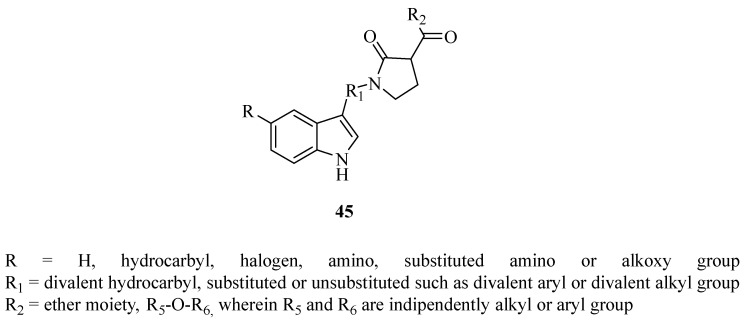
5-Oxopyrrolidines **45** endowed as INIs [137].

The 5-oxopyrrolidines **45** in Figure 17 and bearing the *N-*indol heteroarylcarboxamide scaffold and their structural tautomers are interesting [137]. Such novel compounds are reported as antiviral agents and specifically claimed as novel INIs for the management of HIV-promoted infections [137].

Such compounds do act by inhibiting either the nuclear import of HIV-IN along as well as integration of HIV viral genomic DNA into the host genome [137].

**Figure 18:**
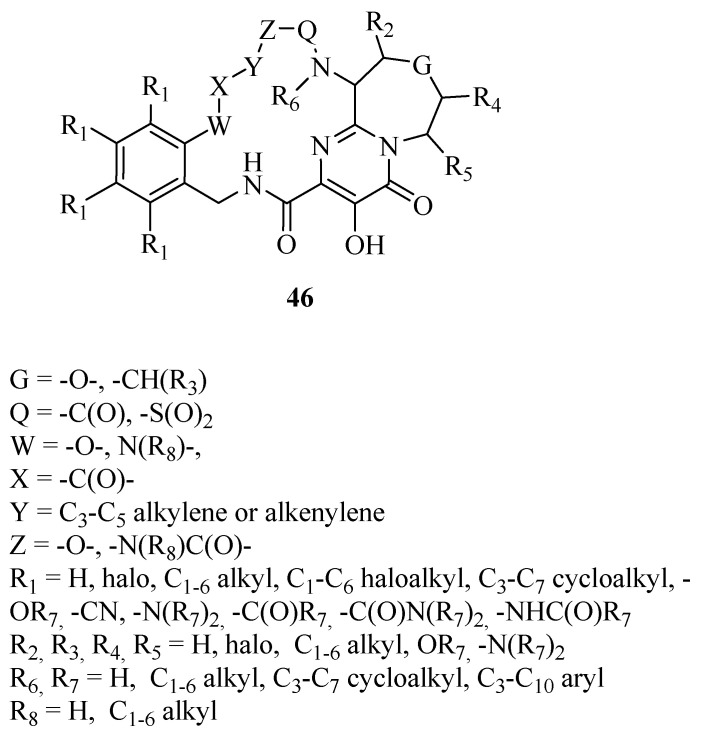
Macrocyclic derivatives **46** [138].

Considering the variegate source of new and effective compounds offered by natural sources, we report a very interesting patent claiming compounds of macrocyclic structure **46** in Figure 18 [138].

## 10. New Strategies against Retrovirus Resistance

After 15 years of cART, there is rising evidence of retroviruses drug resistance which poses a growing threat at a global scale. It is, therefore, imperative that new and effective strategies are needed to improve the therapeutic effectiveness of cART regimens. Such strategies include the discovery of drugs with greater efficacy and higher genetic barriers to resistance than those that are currently used, patient-centered care delivery models, and reliable drug supply chains in conjunction with frameworks for resistance monitoring and prevention [139]. In this context, extended-release treatments play a key role. Long-acting (LA) extended-release injectable formulations or implants represent one of the most important approaches to improving the treatment and prevention of chronic retroviral infection and are critical to the success of these novel delivery mechanisms. These injectable formulations of antiretrovirals (ARVs) represent a viable alternative to improve adherence and effectiveness to retrovirus-associated disease treatment and also for increasing the success of the therapy by combining two or more entities. This approach represents a potentially effective strategy for ultra-LA drug delivery with multiple possible therapeutic applications. LA-ARV formulations, currently in clinical trials, are formulated as nanosuspensions for injections and could open up the way to treat the disease in a different and better way [140,141].

Since the very high replication cycles easily lead to resistance, continuous research efforts are needed to find alternative and efficient therapeutic approaches for the management of retrovirus infections. Experimental approaches of relevance, although still in early stages, include: (i) inhibition of viral DNA integration into the host genome is accomplished by preventing PIC transport into the nucleus. This is put into practice by integrase-mediated nuclear import inhibitors, which bind to the host cell’s nuclear import protein [142]; (ii) IN-mediated chromatin remodeling inhibitors (ICRIs). In this case, inhibition of *v*-DNA integration into the host genome by preventing the opening of the chromatin structure is targeted by the host cell’s chromatin remodeling factors [143].

## 11. Conclusions

The development INSTIs for experimental uses and the introduction into the cART of appropriate derivatives represented a major advancement in the field of retroviruses. These drugs specifically target the viral IN enzyme, which is responsible for integrating the viral DNA into the host cell’s genome during the replication process. INSTIs demonstrated high potency in suppressing viral replication and reducing HIV viral load in infected individuals. In addition, when INSTIs are used in combination with other antiretroviral drugs (i.e., cART), they can effectively control the viral load, allowing patients to lead longer and healthier lives. Compared to some older classes of antiretroviral drugs, INSTIs showed a lower propensity for the development of drug resistance and thus remained effective for a more extended period, reducing the risk of treatment failures. Some INSTIs are available as once-daily dosing, making them more convenient for patients and improving treatment adherence. In addition, INSTIs are often recommended as part of the first-line treatment for HIV-affected patients. Recently have also been used to prevent mother-to-child transmission of HIV during pregnancy and childbirth. Administering these drugs to the mother and newborn resulted in a significant reduction of the risks associated with vertical transmission of the virus.
